# Magnetic Resonance Characterization of Porous Media Using Diffusion through Internal Magnetic Fields

**DOI:** 10.3390/ma5040590

**Published:** 2012-04-12

**Authors:** Hyung Joon Cho, Eric E. Sigmund, Yiqiao Song

**Affiliations:** 1 School of Nano-Bioscience and Chemical Engineering, Ulsan National Institute of Science and Technology, Ulsan 689-798, Korea; 2 Department of Radiology, New York University, 660 First Avenue, New York, NY 10016, USA; E-Mail: eric.sigmund@nyumc.org; 3 Schlumberger-Doll Research, One Hampshire Street, Cambridge, MA 02139, USA; E-Mail: ysong@nmr.mgh.harvard.edu; 4 Athinoula A Martinos Center for Biomedical Imaging, Department of Radiology, Massachusetts General Hospital, Charlestown, MA 02215, USA

**Keywords:** gradients, susceptibility, diffusion, decay due to diffusion in the internal field (DDIF), porous media, trabecular bone

## Abstract

When a porous material is inserted into a uniform magnetic field, spatially varying fields typically arise inside the pore space due to susceptibility contrast between the solid matrix and the surrounding fluid. As a result, direct measurement of the field variation may provide a unique opportunity to characterize the pore geometry. The sensitivity of nuclear magnetic resonance (NMR) to inhomogeneous field variations through their dephasing effects on diffusing spins is unique and powerful. Recent theoretical and experimental research sheds new light on how to utilize susceptibility-induced internal field gradients to quantitatively probe the microstructure of porous materials. This article reviews ongoing developments based on the stimulated echo-pulse sequence to extend the characterization of porous media using both spatially resolved and unresolved susceptibility-induced internal gradients that operate on a diffusing-spin ensemble.

## 1. Introduction 

Magnetic susceptibility contrast-induced inhomogeneous magnetic fields show a fingerprint of the underlying pore geometry when porous media are subjected to an external, uniform magnetic field. Nuclear magnetic resonance (NMR) provides a unique opportunity for nondestructively detecting such internal field variations, and susceptibility contrast-based NMR methods hold great promise for investigating the structural and functional properties of porous media.

For example, fluid-filled rocks, soils, or tissues (e.g., bones), which are comprised of at least two phases with different magnetic susceptibilities, give rise to an inhomogeneous internal magnetic field across their pores when embedded inside a uniform magnetic field. The presence of a nonuniform field often influences NMR-relaxation experiments and diffusion measurements. This effect was first recognized by Brown [1]. Since then, a large amount of effort has gone into understanding the effects of internal gradients on various grain-packing geometries and designing pulse sequences that minimize their effects [2,3,4,5,6]. Recent theoretical and experimental research [7,8,9] sheds light onto utilizing susceptibility-induced internal gradients to probe the detailed structures of porous materials, such as the pore size of oil-bearing rocks and the surface-to-volume ratio of trabecular bones [8,9,10,11,12].

In the context of biological tissues, when a superparamagnetic contrast agent, such as iron-oxide particles [13,14,15,16], is confined to a vascular or blood-bearing space, inhomogeneous and localized fields develop around the capillary vessels due to susceptibility differences between the vessel space, interstitium, and the outside tissue in the uniformly applied magnetic field. The presence of these induced fields may accelerate conventional *T*_2_^*^- and *T*_2_-relaxation rates. As a result, Dennies *et al*. reported that the size of tumor microvessels can be determined by measuring the *in vivo* ratio between susceptibility contrast-induced *T*_2_^*^ and *T*_2_ in the presence of an iron-oxide intravascular contrast agent [17], a technique known as vessel-size imaging. Because the vessel geometry is a significant determinant of susceptibility contrast, in-depth characterization of the internal gradients in the microvessel structure may provide important information about the morphology of abnormal tumor vessels [18,19,20].

The sensitivity of NMR for measuring internal field variations led to the development of several pulse sequences to characterize them. Traditionally, the signal of the gradient echo is reduced below the spin-spin relaxation limit due to dephasing of the magnetization from the inhomogeneous field, which is known as ${T_{2}}^{\prime }$ relaxation. Therefore, asymmetric spin-echo sequences, which sample the magnetization away from the point of maximum spin coherence, are sensitive to effects of susceptibility contrast [21,22]. The decay due to diffusion in the internal field (DDIF) method uses a stimulated echo sequence and the fact that the internal field, which is modulated by spatial variations in susceptibility, causes signal decay when sampled by a diffusing spin ensemble. DDIF essentially monitors the time-dependent evolution of magnetization in the internal magnetic field and determines the distribution of its decay constants in order to obtain quantitative information on the pore structure. The structural parameters of a fluid-filled porous medium, such as the pore size of oil-bearing rocks and the surface-to-volume ratio of a network of trabecular bones, can be estimated by the decay characteristics of magnetization in the presence of internal gradients using bulk DDIF contrast [7,8,9].

In this paper, we will review several recent extensions of the DDIF method that use microimaging to verify that the strength and orientation of the susceptibility-induced internal gradients can be faithfully mapped using stimulated echo-based NMR relaxometry [8,23,24,25]. When the pore size is larger or comparable to the image voxel, spatially resolved DDIF contrast provides detailed information about the strength and orientation of the internal gradients by measuring susceptibility contrast across the pores. For smaller pores, the variation in the internal gradient may be unresolved because the signals from multiple pores are averaged across a single image voxel. However, even in this case, DDIF-encoded relaxation data still contains salient structural information that can be inferred using proper analytical techniques, such as Laplace inversion or spectrum heterogeneity [9,26].

An alternative quantitative description of the internal field can be obtained through self-correlation of the field. This approach is useful when the pore size of the underlying microstructure is significantly smaller than the size of the magnetic resonance imaging (MRI) voxel, which is the case for fine-grain rocks and tissue microvessels. In these cases, the statistical properties of the internal field may be used to determine relevant structural parameters. For example, Audoly *et al*. showed that the structural factor (*i.e*., 2-point density correlation function) of porous media can be approximated using the self-correlation function of the internal magnetic field [27]. They further argued that this correlation is closely related to the fluid transport properties of the material. Cho *et al*. demonstrated that a pulsed-field gradient (PFG) method involving both applied and internal field gradients, can be used to obtain spatial magnetic correlation functions that are in good agreement with theoretical simulations [28]. For biological applications, such as brain imaging, Jensen *et al*. showed that temporal magnetic field correlation (MFC) functions can be measured using the asymmetric spin-echo sequence with a range of asymmetric time shifts followed by Gaussian fitting. They further argued that MFC is a more specific metric of the microscopic field inhomogeneities compared with conventional relaxation parameters, such as *T*_2_ or *T*_2_^*^ [21,22]. Thus, a range of techniques and applications exist that use internal fields to increase their microstructural sensitivities.

This article is organized as follows: first, the basic concept of stimulated echo-based internal field contrast will be introduced along with its extension to an imaging module, followed by the basic data-analysis method used for bulk (*i.e*., spatially unresolved) and imaging experiments. DDIF imaging experiments that use a 2-dimensional (2D) cylindrical phantom will be discussed to show the direct correlation between spatially resolved DDIF contrast and the strength of a local susceptibility-induced internal gradient [23]. Furthermore, the feasibility of measuring the local axis of orientation of the cylindrical microstructure from directional measurements of its internal gradient distribution will be discussed. Illumination of the origin and the contrast mechanism that were previously reported for porous media, such as trabecular bone and which complement the interpretation of the bulk DDIF technique, will be reviewed along with spatially resolved DDIF. Finally, a pulsed-field gradient-NMR (PFG-NMR) method that utilizes an asymmetric, stimulated echo sequence to extract the magnetic field correlation function will be discussed, describing the connections between the statistical properties of the internal field and a porous material’s structural 2-point correlation function. Representative examples in a system of randomly packed beads will be described using a pair of symmetric and asymmetric stimulated echoes [28]. Finally, these examples will be discussed in the broader context of microstructurally sensitive MR techniques, and future applications will be considered.

## 2. Utilization of the Internal Magnetic Field for Studying Porous Media

### 2.1. Diffusion in the Presence of Internal Field Gradients

NMR measurements of the apparent diffusion coefficients (ADC) of molecules have provided unique structural details on porous media since the advent of PFG-NMR [29,30]. Once spatially encoded with a magnetic field gradient, diffusing molecules give rise to an effective signal reduction of the NMR spin echoes, which enables an accurate estimation of ADC with given known field gradients. In addition to their absolute values, directional anisotropy of ADC values has been observed and provides useful structural contrast in porous and biological media [31,32]. It has also been theoretically and experimentally shown that the time-dependent diffusion coefficient of porous media contains information about the surface-to-volume ratio [33,34]. 

The influence of the internal magnetic field gradients generated by variations in magnetic susceptibility on NMR-diffusion measurements has also been recognized. If the internal gradient significantly interferes with the external PFG gradients, the apparent extracted diffusivity can be altered. Various methods have been suggested as ways to overcome this interference [3,4,5]. On the other hand, susceptibility-induced internal gradients contain the fingerprint of the underlying structures, and thus several techniques that focus on quantifying the effects of diffusion within internal gradients alone have been developed to extract such properties as the pore size or the surface-to-volume ratio from porous media [9,35,36,37]. In this review, we focus on describing the decay of the echo signal through diffusing molecules in the presence of internal gradients (and in some cases external gradients) using stimulated echo-based pulse sequences. As we will discuss below, the translational motion of the spins is a vital component of these techniques, although the diffusivity itself is sometimes an implicit parameter rather than an explicitly measured quantity.

**Figure 1:**
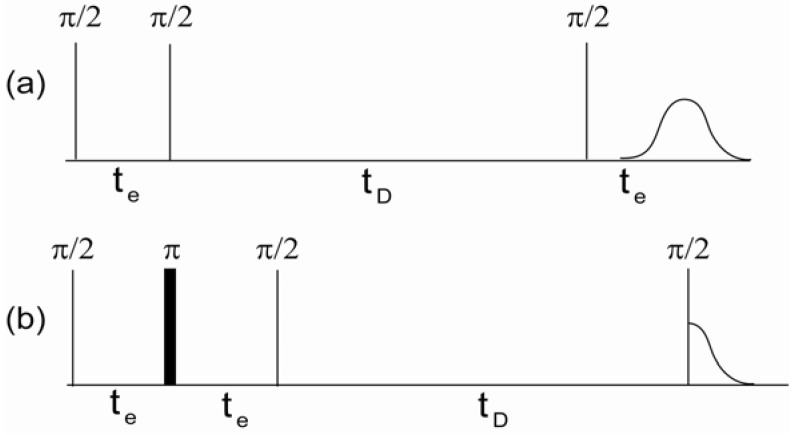
(**a**) Decay due to diffusion in the internal field (DDIF) and (**b**) reference pulse sequences. $t_{e}$ and $t_{d}$ are the encoding and diffusion time periods, respectively. Stimulated echoes were detected by DDIF and free induction decay for the reference scan. Reproduced from Reference [12] with permission.

DDIF is a stimulated echo-based relaxation measurement that utilizes susceptibility-induced internal magnetic field gradients and molecular diffusion to probe the structures of porous media [7,9]. Imagine that the magnetic field, $B_{z}$ is higher in one part of the pore and lower in another due to the susceptibility contrast of the medium. The first step of DDIF is to establish a spatial magnetization profile that represents the inhomogeneous local magnetic field across the pore. A schematic DDIF pulse sequence is shown in Figure 1a. Transverse spin magnetization after the first $\frac{\pi }{2}$-pulse develops a spatially dependent phase due to variations in the local internal magnetic field during the encoding period ($t_{e}$). At the end of the encoding period, the encoded transverse magnetization is flipped to the longitudinal direction and the spins diffuse during the diffusion period ($t_{d}$). This movement of diffusing spins across different regions of the internal magnetic field causes a reduction in the magnetization profile. This reduction in the magnetization can be detected using a third $\frac{\pi }{2}$-pulse, which produces a stimulated echo. The decay of these amplitudes typically demonstrates multi-exponential decay behavior for variable diffusion times ($t_{d}$) due to the distribution of field gradients in the sample. When the molecules diffuse a distance ($l_{D}=\sqrt{2Dt_{d}}$) equal to the pore size, the spatial nonuniformity of the magnetization diminishes, and the magnetization decay is dominated by spin-lattice relaxation (*T*_1_). Figure 1b shows the pulse sequence used to measure the spin-lattice relaxation times as a reference experiment to DDIF contrast. The π-pulse in the middle of the encoding period unwraps the spatially dependent phase acquired during $t_{e}$ due to the inhomogeneous magnetic field and re-establishes a uniform magnetization. This suppresses the effect of the inhomogeneous field on the variable $t_{d}$, while retaining the same spin-spin (*T*_2_) relaxation present in the DDIF-encoded sequence for convenient normalization. The combination of DDIF and reference scans allows a stronger distillation of the internal field contrast from the total magnetization.

**Figure 2:**
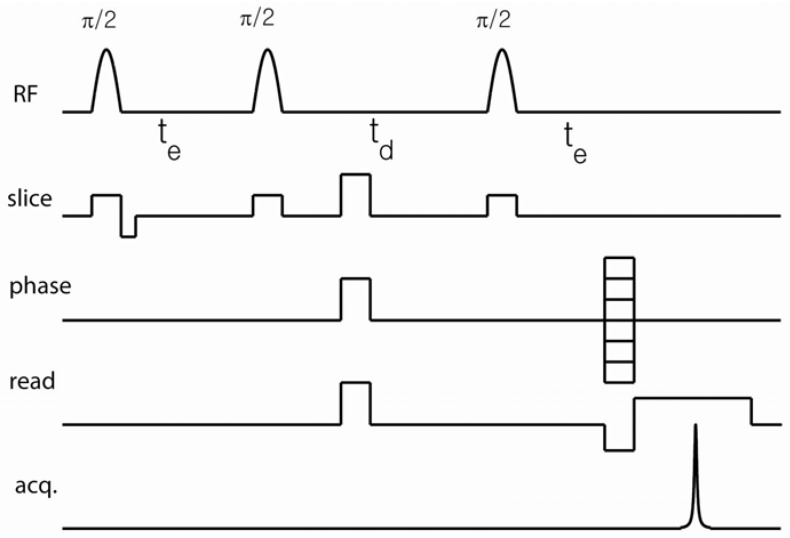
Slice-selective 2-dimensional (2D) spin-warp DDIF imaging sequence. The spoiler gradient was applied during $t_{d}$ to remove the unwanted coherence pathway signal. The readout gradient was applied just before stimulated echo acquisition to reduce signal decay due to the external field gradients at different diffusion times. A pulsed-field gradient can be applied during $t_{e}$ to extract cross-terms between the internal and external gradients. Reproduced from Reference [23] with permission.

Extensions of DDIF contrast, including its use in imaging modules, can be implemented in order to obtain spatially resolved information regarding the distribution of the internal field gradient. In this article, we review its extension to 2D spin-warp imaging, as shown in Figure 2, but the basic DDIF pulse sequence can be attached as a precursor to other imaging modules. In such a sequence, the DDIF pulse sequence encodes the magnetization contrast and an imaging technique is applied to observe its spatial distribution. The concept of DDIF imaging is thus similar to that of *T*_1_-, *T_2_*-, and diffusion-weighted imaging. In particular, the stimulated echo sequence can precede any fast imaging modality, such as RARE or BURST, to accelerate the acquisition of spatially resolved DDIF contrast [38]. In the case of spin-warp imaging, as shown in Figure 2, field gradients are applied during soft $\frac{\pi }{2}$-pulses for slice selection. The use of pulsed spoiler gradients during $t_{d}$ ensures the selection of the stimulated echo pathway. Read and phase gradients are applied just before the stimulated echo formation to minimize unwanted diffusion-weighting from these gradients.

Because applied field gradients also cause signal decay, one needs to be cautious during the design of pulse sequences to minimize any encoding gradients before $t_{d}$ because they will interfere with internal field encoding. Read and phase gradients can occur after $t_{d}$, and they induce, at most, $t_{d}$-independent diffusion-weighting. However, an unfocused slice gradient during the second $\frac{\pi }{2}$-pulse or a crusher gradient before the second $\frac{\pi }{2}$-pulse can induce $t_{d}$-dependent diffusion-weighting and interfere with the internal gradient during $t_{d}$. For example, the effective decay rate of the slice-selective gradient of the second $\frac{\pi }{2}$-pulse (see Figure 2) can be estimated according to the equation, $\frac{1}{T_{\text{eff}}}=\gamma^{2}{\left(\frac{G_{s}}{2}\right)}^{2}{\tau_{p}}^{2}D$, where, *τ*_*p*_, *G_s_* and *D* correspond to the pulse length, slice-gradient strength, and diffusivity, respectively. γ is the gyromagnetic ratio. When *τ*_*p*_ = 2 ms and *G_s_* = 4 G/cm, this estimation produces an additional decay rate of 0.3 (1/s). The total background decay rate of the DDIF imaging sequence in Figure 2 can be estimated as $\frac{1}{T_{\text{eff}}}+\frac{1}{T_{1}}=0.7$ (1/s), assuming the *T*_1_ relaxation of free (unrestricted) water molecules. This correction is one example of the need to separate internal and external field effects when interpreting DDIF data. Future examples in this review will carry this idea further and illustrate the constructive and destructive interference patterns of the internal and external gradients.

### 2.2. Data Analysis

Before explaining the appropriate data-analysis techniques used to analyze bulk and imaging DDIF contrast, it is worthwhile to introduce the length scales relevant to the DDIF experiment: imaging voxel size (Δx), structural scale (*l_s_*), and diffusion length (*l_D_*). The structural scale (*l_s_*) refers to the average pore size of the medium, and the diffusion length (*l_D_*) is defined as, $l_{D}=\sqrt{2Dt_{d}}$ where *D* is the diffusion constant and $t_{d}$ is the diffusion time, respectively [39,40]. 

For bulk DDIF measurements, when Δx >> *l_s_*, *l_D_*, the decay of the echo amplitudes typically shows multi-exponential behavior for short $t_{d}$, reflecting the internal gradient distribution in the sample. In these cases, the DDIF signal is essentially a sum of weighted exponential functions (*i.e*., a Laplace transformation), as given by Equation (1).

(1) $$S(t)=\int f(\tau )e^{-t/\tau }d\tau$$

**Figure 3:**
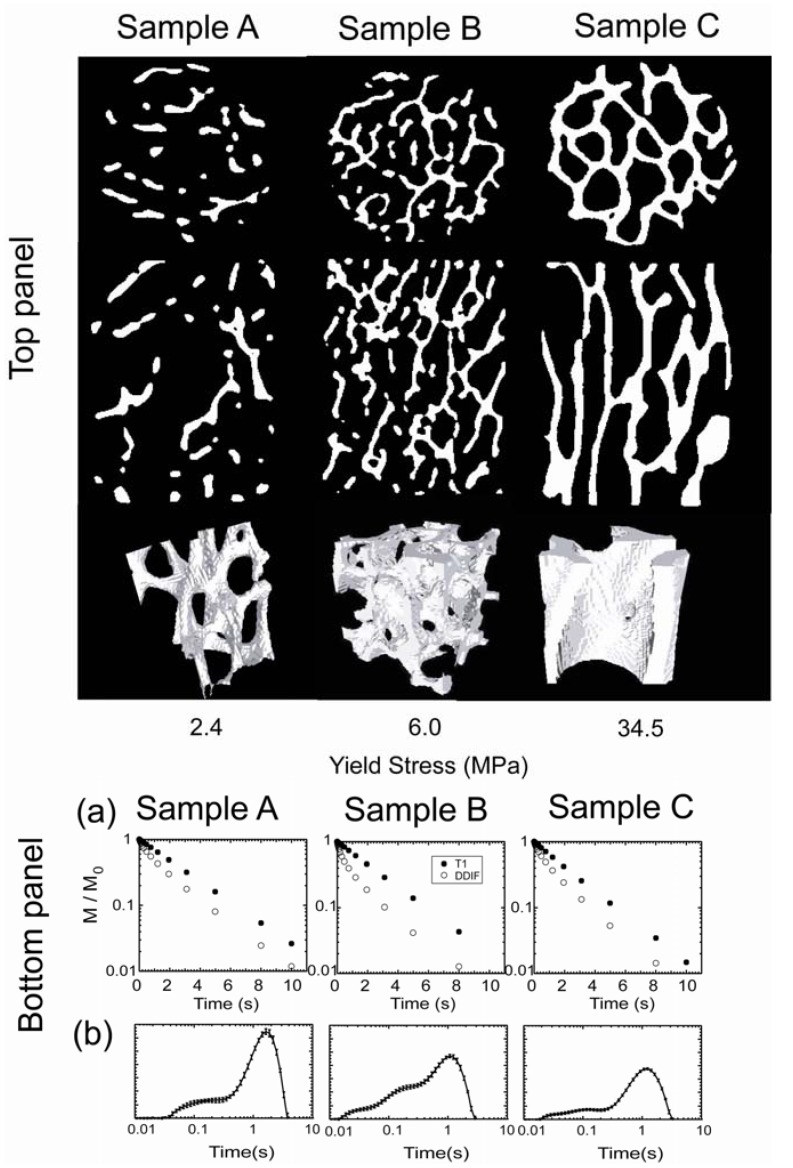
In the top panel, high-resolution microscopic computed tomography (μCT) images of 3 trabecular bone samples with different yield stresses are shown. Transverse and longitudinal slices are shown in the first and second rows, respectively. A 3D rendering of a cubical subvolume is shown in the third row. Images were acquired at an isotropic spatial resolution of 34 µm. In the bottom panel, section (**a**) shows the corresponding decay in the raw echo amplitudes as a function of the diffusion time of each sample. Section (**b**) shows the corresponding DDIF spectra obtained from Laplace inversions. Reproduced from Reference [12] with permission.

As $t_{d}$ grows longer, the echo amplitude eventually follows regular spin-lattice relaxation as the spatial nonuniformity of magnetization disappears. To illustrate this behavior, representative DDIF and reference (*T*_1_) data on 3 different bovine trabecular bone samples are shown in the bottom panel of Figure 3a. The difference in magnetic susceptibility between the solid and liquid phase of a water-filled trabecular bone gives rise to field variations and contributes to DDIF signal decay over short $t_{d}$. The corresponding trabecular structure of each sample is shown in the top panel, obtained from high-resolution microscopic computed tomography (μCT). Qualitatively, a faster initial decay rate is apparent in the time domains of the DDIF echo amplitudes of sample B with respect to samples A and C. In the bottom panel of Figure 3b, the corresponding DDIF spectra are shown for each sample [12].

To obtain the DDIF spectra, Laplace inversions are performed on the echo amplitudes that are acquired at logarithmically spaced $t_{d}$. Reference data (*R*) are subtracted from the DDIF data ($E_{0}$) to remove the relaxation contribution, as shown in Equation (2).

(2) $$E_{s}=E_{0}-a_{0}R$$

The Laplace inversion procedure is then applied to the subtracted amplitudes ($E_{s}$) to obtain the spectra of the decay times. Since an individual inversion can be numerically ill-conditioned, proper consideration of noise sensitivity is helpful. The uncertainty of the DDIF spectrum can be verified using a number of Laplace inversions of the original data combined with different simulations of the Gaussian-distributed noise, whose amplitude matches the measured echo amplitudes standard deviation. Inversion consistency can be maintained by using a constant regularization parameter for each data set. As shown in the bottom panel of Figure 3b, the DDIF spectra clearly show larger weights for the short, fast decay times of sample B, which is consistent with the faster decay of its time-domain DDIF decay signal shown in Figure 3a [12].

Conversely, when the voxel size of interest is much smaller than the relevant structural length scale (Δx << *l_s_*), a constant gradient within each high-resolution voxel may be assumed and the decay of the echo amplitude of each imaging voxel can be fit to a single exponential model. Consequently, the echo decay rate of each voxel can be modeled as shown in Equation (3) [41,42].

(3) $$\frac{1}{T_{\text{DDIF}}}=\frac{1}{T_{\text{back}}}+\gamma^{2}G_{{\mathrm{int}}^{2}}te^{2}D$$

Equation (3), *T*_back_ is the background relaxation time, including *T*_1_ and diffusion-weighting of the slice gradient, *γ* is the gyromagnetic ratio, *G*_int_ is the strength of the internal gradient, $t_{e}$ is the encoding time, and *D* is the diffusion constant. Note that this formula omits directionally dependent cross-terms between the internal and external gradients; this effect will be discussed in upcoming sections. In order to experimentally extract the DDIF decay rates affected by the internal gradients, these background rates can be subtracted after fitting a single exponential function to the individual pixels of the image. It is worthwhile to estimate the noise level of the signal-free region of the image and include only data points with amplitudes larger than the noise level. In the DDIF-imaging experiment, it was also observed that the signal decay that was obtained at low resolution, in some cases, demonstrated multi-exponential behavior when there was a significant gradient distribution within each voxel. Ideally, Laplace inversions could be applied to the data in each voxel; however, voxel-wise data sets may not be sufficiently sampled in time ($t_{d}$) or possess insufficient signal-to-noise ratio (SNR) to support Laplace inversions. In these cases, reasonable comparisons can be made by limiting the fit to a shorter diffusion time over which most of the voxels display single exponential behavior. This initial rate represents the overall average rate of the entire voxel.

### 2.3. DDIF Microimaging with a 2D Cylindrical Phantom

Some varieties of porous media (e.g., vascular capillary bundles, axonal nerve fibers, myofibers) possess some degree of symmetry and collective anisotropy, which are closely related to their integrities and functions. In some cases, DDIF may be employed to sensitize MR to microstructural anisotropy via its influence on the internal field pattern. Furthermore, in the simplest case, the cylindrical symmetry of a bundle of capillary tubes (*i.e*., “fibers”) makes it essentially a 2D object, so that exact comparisons with theoretical calculations can be performed. When intrinsic or extrinsic susceptibility contrast exists between the fiber and the surrounding medium, internal gradients arise at the interface around the fiber. Quantitative characterization of these gradients may provide important tools to infer the fibrous microstructure, such as the directions of the nerve pathways in brain, cardiac or skeletal muscle architecture, or the tortuosity of cancerous microvasculature. To that end, techniques can be employed to offset the internal and external gradients and maximally sensitize DDIF to anisotropy.

**Figure 4:**
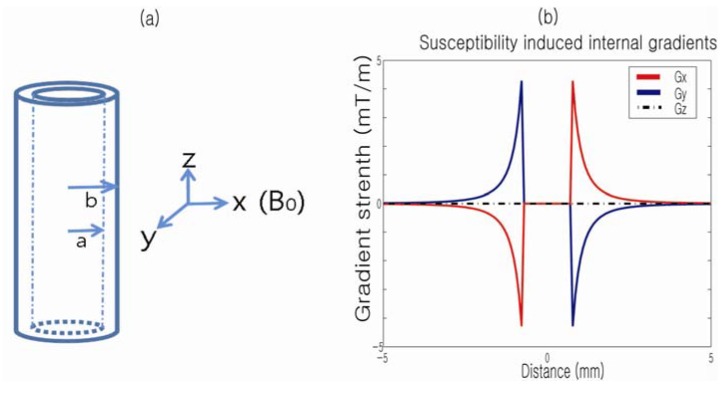
(**a**) Capillary cylinder in the presence of *B*_0_ (diameter: 1.55 mm; wall thickness: 0.2 mm); (**b**) Calculated susceptibility-induced internal gradients along the *x*, *y* and *z* directions. Reproduced from Reference [43] with permission.

The susceptibility-induced internal gradient profile of a single capillary tube in the presence of a magnetic field, *B*_z_, along the axial direction (*z*) of the cylinder offers insights into the anisotropy of the internal field (Figure 4). The difference in susceptibility (Δχ) between the wall of the cylinder and the rest of the medium is the source of the internal gradient and can be analytically described as shown in Equation (4).


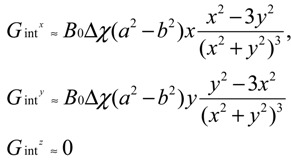
(4)

Figure 4b plots the profiles of the corresponding susceptibility-induced gradients along the *x*, *y* and *z*-axes, respectively. Note that no internal gradients exist when the field is applied along the *z*-axis of the cylinder. Also, for a transversely applied field, the internal gradient is oppositely oriented on either side of the cylinder and is absent within it.

**Figure 5:**
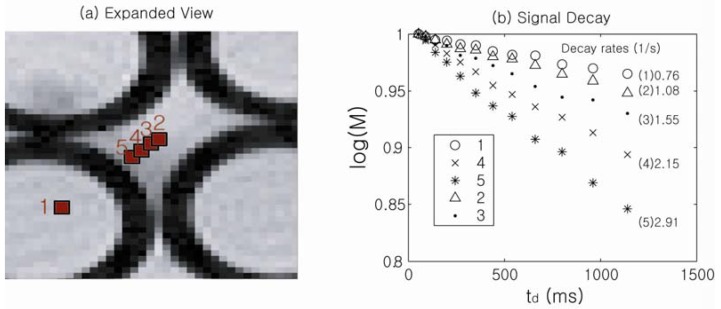
(**a**) Spin-density image of the pore space in packed cylindrical capillary tubes; (**b**) Signal decay rates of each point labeled in (**a**). Reproduced from Reference [23] with permission.

In order to experimentally obtain spatially resolved information on the susceptibility-induced internal gradients, a DDIF-imaging experiment was performed. Figure 5 shows an expanded spin-density image of a randomly packed water-filled glass capillary phantom (inner radius: 0.58 mm, outer radius: 0.77 mm), and Figure 5b shows the corresponding DDIF signal decay of the individual voxels denoted in Figure 5a. The details of this acquisition have been previously described [43]. The susceptibility-induced internal field is approximately constant inside the cylinders, and DDIF decay rates of 0.76 (1/s) for point 1 inside the cylinder is in good agreement with the sum of background contributions from *T*_1_ (0.4 (1/s)) and the uncompensated slice gradients (0.3 (1/s)). 

**Figure 6:**
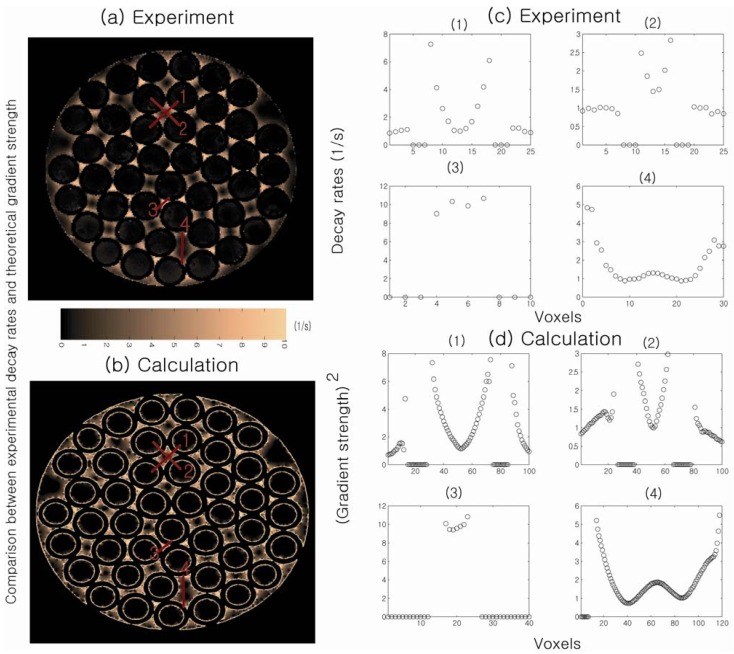
(**a**) Measured DDIF decay rates in packed cylindrical capillary tubes; (**b**) Calculated strength of the internal gradients in the corresponding pore space; (**c**) Experimental measurements of DDIF decay rates along the lines across the pores denoted in (**a**). (**d**) Theoretical calculation of the squared internal gradient across the lines in the same pore space denoted in (**b**). Reproduced from Reference [23] with permission.

Figure 6 shows a direct comparison between spatially resolved DDIF rates and theoretically calculated internal gradient values at corresponding positions in the randomly packed capillary tubes. The signal decay rate is slower at the center of the cylinder and at the extracylindrical pore center and faster near the walls, indicating higher internal gradients near the walls of the cylinder. Agreement between the theoretical calculations of the internal gradients and the DDIF measurements is excellent. This work demonstrates the sensitivity of DDIF imaging is sufficient to directly characterize susceptibility-induced internal gradients.

It is worthwhile to note that the DDIF-imaging method essentially measures the squared value of the local internal gradient (*G*_int_^2^), as shown in Equation (3). As a result, any directional information regarding the internal gradient is lost. In general, the gradient of the magnetic field vector is a second-rank tensor. In the presence of a strong magnetic field along the *z*-axis, only 3 partial derivatives ($\frac{\partial B_{z}^{i}}{\partial z}$, $\frac{\partial B_{z}^{i}}{\partial x}$, $\frac{\partial B_{z}^{i}}{\partial y}$) of the *z*-component of the susceptibility-induced magnetic field ($B_{z}^{i}$) are of interest because the others ($\frac{\partial B_{x}^{i}}{\partial z}$, $\frac{\partial B_{y}^{i}}{\partial z}$, …) correspond to fields in the rotating frame and do not affect the long-term spin dynamics. As a result, three partial derivatives characterize the relevant internal gradient for NMR measurements of an isotropic-susceptibility constant (*χ*). Because the internal magnetic field gradient is a vector quantity, not only the strength but also the orientation of the internal gradient should provide the maximum amount of information about the underlying structural geometry. Conventional DDIF measurement, as illustrated thus far, only reflects the magnitude of the gradient, not the orientation. 

To determine the orientation of the internal gradient, an external field gradient can be applied to generate a cross-term between the external and internal gradients. Let us imagine that a pulsed-field gradient of arbitrary direction is applied during encoding period ($t_{e}$) of the pulse sequence, as shown in Figure 2. The spins experience both internal and external gradients during the encoding period and, thus, magnetization decays following a new, effective decay rate ($\frac{1}{T_{\text{cross}}}$) as the diffusion time ($t_{d}$) increases.

$$\frac{1}{T_{\text{cross}}}=\frac{1}{T_{\text{back}}}+\gamma^{2}C_{{\text{total}}^{2}}t_{e^{2}}D$$

(5) $$G_{\text{total}}^{2}=G_{\mathrm{int}}^{2}+G_{\text{ext}}^{2}+2{\vec{G}}_{\mathrm{int}}\cdot {\vec{G}}_{\text{ext}}$$

The squared magnitude of this gradient ($G_{\text{total}}^{2}$) contains a cross-term (vector dot product) of the internal and external gradients, as shown in Equation (5). The orientation of the internal gradient in an image voxel can be estimated by varying the interference effects of the internal (${\vec{G}}_{\text{int}}$) and external (${\vec{G}}_{\text{ext}}$) gradients on the magnetization decay as the direction of the external gradient is changed. In fact, three measurements with independent, external field gradient directions, and one without an external gradient, are sufficient for determining the local ${\vec{G}}_{\text{int}}$ vector within an imaging voxel according to Equation (5).

**Figure 7:**
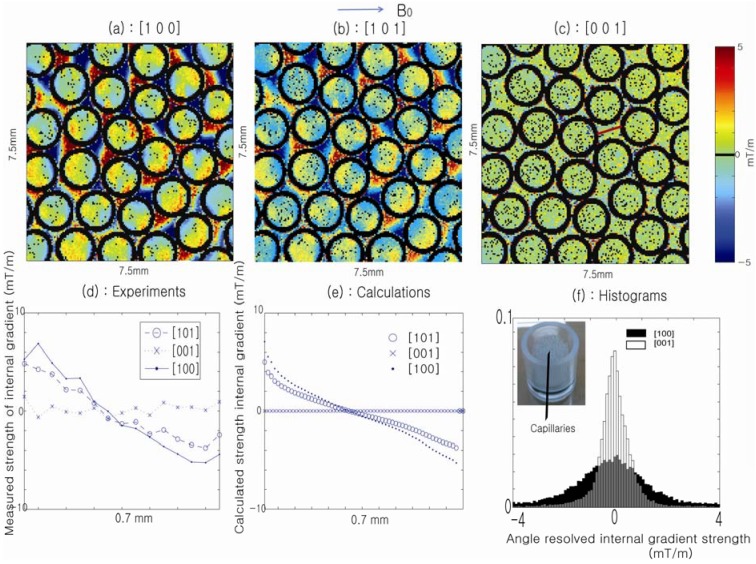
In the top panel, sections (**a**–**c**) show spatial maps of the DDIF cross-term rates, respectively, when external pulsed-field gradient (PFG) is applied along different directions. In the bottom panel, sections (**d**) and (**e**) compare the internal gradient strength across the pore denoted in (**c**) determined from experimental DDIF rates with that derived from theoretical calculations. (**f**) Histogram distribution of the measured internal gradient strengths along the [100] and [001] directions, respectively. Reproduced from Reference [43] with permission.

Figure 7 illustrates the significant changes that occur in the spatially resolved DDIF rates as the direction of the applied field gradient (5 mT/m; which is on the order of the internal gradient used in this experiment) is changed from an axial to a transverse orientation relative to the cylindrical axis. When the external gradient is applied perpendicularly to the cylindrical axis (a), the fastest decay rates are observed in the regions where the internal and external gradients have the same direction. When the external gradient is applied parallel to the cylindrical axis (c), no apparent effect on the cross-term is observed because no internal gradient is present along the direction of the cylindrical axis. In the bottom panel, comparisons between the experimental and theoretical calculations and histogram analyses of the decay rates for both cases are shown to illustrate the differences in the distributions of the decay rates when the direction of the external gradient is varied. The absence of an axial component in the internal gradient suggests the feasibility of determining the direction of the cylindrical capillaries using the cross-term between the external and internal gradients, similar to recent findings [43].

**Figure 8:**
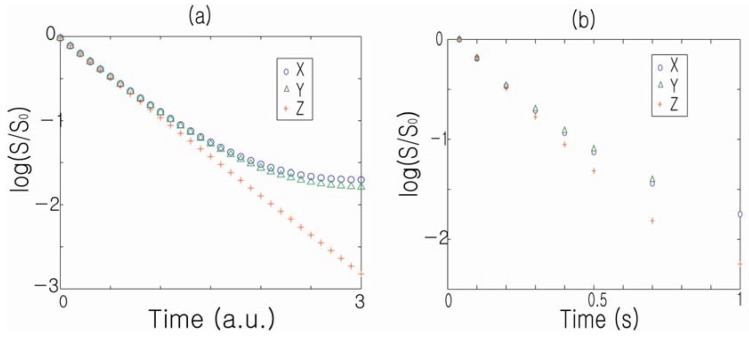
(**a**) Calculated decay characteristics of the unresolved DDIF signal when the external gradient is applied along the *x*, *y* and *z* directions, respectively; (**b**) Corresponding experimental measurements of the DDIF-signal decay. Reproduced from Reference [43] with permission.

It is worthwhile to note that internal gradients may contribute to non-Gaussian diffusion-weighted behavior. For example, in the case of a 2D cylindrical phantom, the diffusion-weighted signal follows a single exponential temporal decay when the external gradient is applied along the *z*-direction. On the other hand, a clear deviation from single exponential behavior is observed when the external gradient is applied along either the *x*- or *y*-direction, where broad distributions of internal gradients exist, as shown in Figure 8 [43]. Capuani *et al*. also recently reported spatiotemporal anomalous diffusion in heterogeneous porous media and hypothesized that the internal gradients act as contributing factors [44]. While different control variables (diffusion time, applied diffusion weighting) may have been applied in these examples, the underlying mechanism of internal gradients inducing apparent non-Gaussian diffusion is a potential common theme.

### 2.4. Comparison between Bulk and Resolved DDIF Contrast in Trabecular Bone

Spatially resolved internal gradient profiles of porous media provide rich information regarding the underlying structures and complement conventional DDIF-relaxation measurements. For example, in this review, we compared bulk and spatially resolved DDIF contrast in trabecular bone. 

Bone tissue is a solid mineral matrix, filled with soft bone marrow that is comprised of a mixture of liquid and adipose (fatty) tissue. Human bone is normally classified as cortical or trabecular. Cortical bone, which usually comprises the shaft of a long bone, is very dense with low porosity. Trabecular bone (spongy bone) is a complex network of plates and rods with a typical thickness of the order of 100 µm that occurs for example in vertebral bodies, and femoral, or tibial joints. These load-bearing zones are often at high risk of fracture in patients with abnormal bone remodeling, such as those with osteoporosis. Total bone density is commonly accessed with dual X-ray absorptiometry (DEXA), which senses the amount of bone without regard to its microscopic arrangement. The bone microstructure of trabeculae can be characterized using X-ray-based μCT; however, the high dose of radiation necessary for current X-ray technology, limits its clinical application. Because MRI provides superior soft tissue contrast without ionizing radiation, there is a great deal of interest in developing clinical MRI methods that can be used to probe the trabecular bone structure *in viv*o; in that spirit, methods that employ the internal field signature via its effect on free induction decay, as well as direct microimaging of the trabecular bone structure in certain anatomical zones, are gaining prominence [45,46,47,48,49,50,51,52,53,54,55,56].

Because the susceptibility difference between the solid bone matrix and the intervening composition of fluid, marrow, and fat gives rise to magnetic field variations in trabecular bone, several MRI methods were developed to characterize the relationship between the strength and linewidth (1/*T*_2_') of bone tissue. Several studies consistently show a prolonged *T*_2_' value in osteoporosis patients that increases as the intertrabecular space reduces the surface area where the susceptibility-induced field exists.

**Figure 9:**
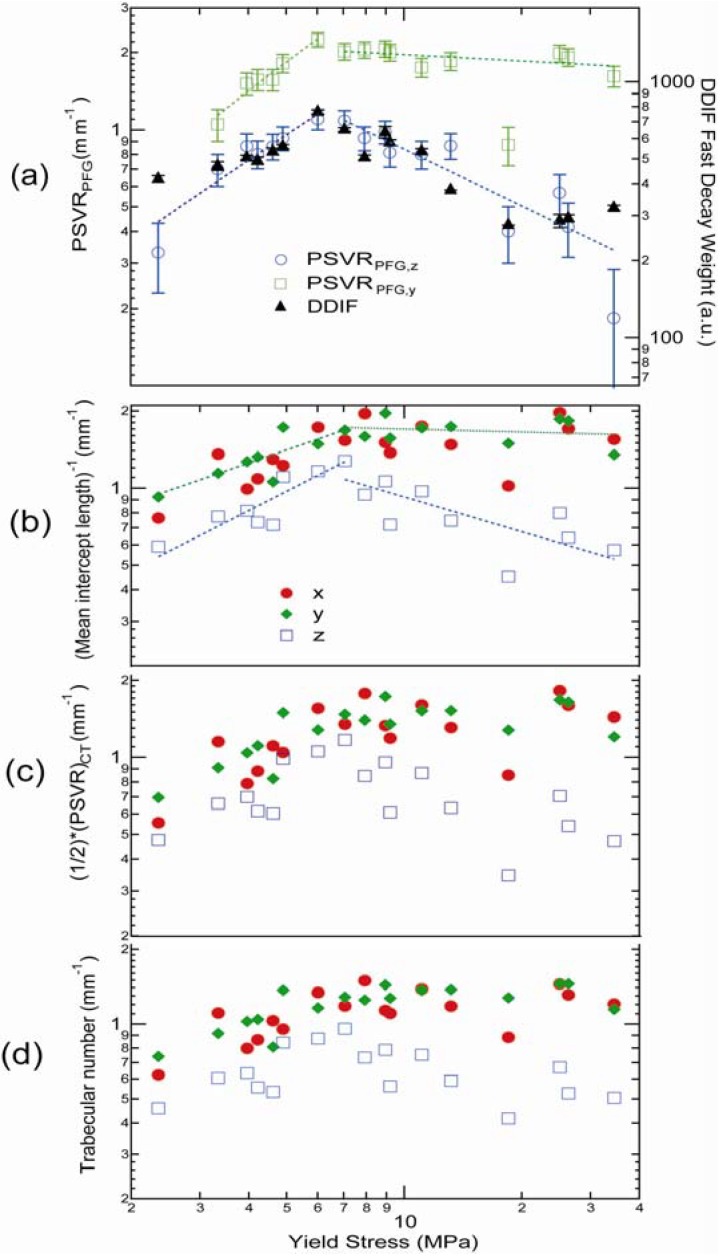
(**a**) DDIF results and PFG-PSVR measurements as a function of yield stress for 17 trabecular bone samples; (**b**) Calculated mean intercept lengths of the corresponding samples. (**c**) Calculated projected surface-to-volume ratio (PSVR) values of the μCT images. (**d**) Calculated trabecular number for corresponding sample. Reproduced from Reference [12] with permission.

Research on bulk-DDIF measurement of bovine trabecular samples [12] (Figure 3 and Figure 9) shows higher spectral weights in short-decay regions of bone samples with high surface-to-volume ratios. For the full range of samples examined in that study, it was shown that the integrated weight of the short-decay region (20 ms < *T*_DDIF_ < 500 ms) of the DDIF spectra of each sample correlates well with the projected surface-to-volume ratio (PSVR) that was determined using both independent PFG-NMR measurements as well as the mean intercept length (MIL) of the trabecular bone samples obtained using μCT image analysis, as shown in Figure 9. MIL was found by averaging the lengths of the line segments between trabeculae across the whole structure. MIL and other related metrics, such as trabecular density, are well-known measures of the integrity of trabecular bone structures. 

The concept of PSVR is similar to that of ADC in an anisotropic medium along the direction of the applied gradient. The leading order behavior of the time-dependent diffusion coefficient in an anisotropic medium (*D*(*t*)) is described by $\frac{D(t)}{D_{0}}\approx 1-\frac{4}{3\sqrt{\pi }}{\left(\frac{S}{V}\right)}_{p}\sqrt{D_{0}t}$, where ${\left(\frac{S}{V}\right)}_{p}$ corresponds to PSVR along the direction of the applied external gradient [33].

To independently measure PSVR using NMR, in comparison with DDIF measurements, the time-dependent diffusion coefficient (*D*(*t*)) was measured using PFG-NMR on the same trabecular bone samples along different directions of the applied external gradient. PSVR was obtained from the slope of the *D*(*t*) data, as suggested by Mitra *et al.* [33]. The MIL values of each sample were found by drawing a line in the proposed direction through the 3D image volume and averaging the distances between trabecular plates along the line of that orientation through the whole sample volume. In Figure 9a, PSVR measurements of the time-dependent diffusion measurements and the corresponding DDIF weight for short-decay times are plotted against the mechanical yield stress for the full range of samples. DDIF weight is seen to closely resemble PSVR measurements along the *B_z_*-direction. In Figure 9b, Figure 9c and Figure 9d, calculated PSVR, inverse MIL and trabecular number from high-resolution (34 µm) μCT images of the same bone samples are plotted to show the close correlation between DDIF measurements and PFG-NMR measurements. Direct calculation of the internal magnetic field of the trabecular bone samples also sheds some light on the origin of DDIF contrast. We noted that deviations in the internal magnetic field from the applied field mainly occurred near plates that were oriented perpendicular to the applied field (*B_z_*) direction.

**Figure 10:**
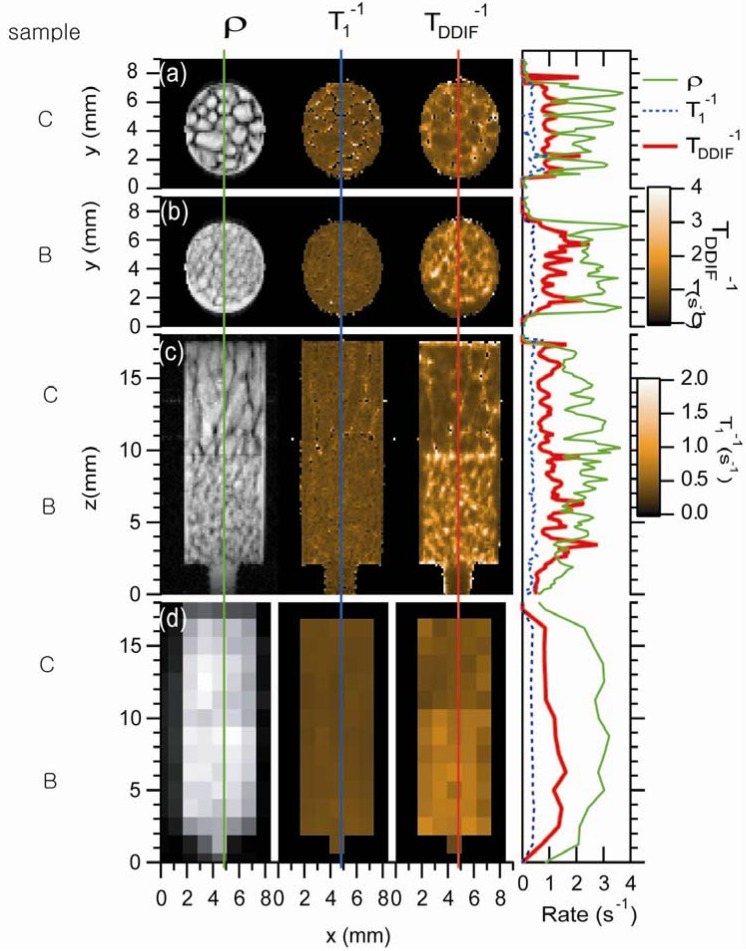
Experimental DDIF microimaging. Spin-density images are shown in the first column. The second column shows a map of the spin-lattice relaxation rate. The third column shows a map of DDIF decay rates. The fourth column shows 1D profiles through the centers of each image, as indicated by the vertical lines. The first and second rows show axial images of samples C and B, respectively. The third row shows high-resolution coronal images of both samples B and C together, and the fourth row shows low-resolution coronal images of samples B and C. Reproduced from Reference [24] with permission.

Spatially resolved DDIF experiments with high (125 µm) and low (1.2 mm) resolution images of the trabecular bone samples also provide direct insight into DDIF contrast [24]. Figure 10 shows the experimental micro-imaging results of trabecular bone samples B and C [24]. Three different types of images—a spin-density image, a 1/*T*_1_ rate map, and a DDIF rate map—of four different cases are plotted: two axial images of two samples, one coronal image intercepting both samples, and one low-resolution coronal image. One-dimensional profiles of spin density and DDIF decay rates were extracted for quantitative comparison, as shown in Figure 10. For both samples and orientations, clear DDIF decay rates maxima and spin-density minima were observed near the trabecular surfaces. Spatially resolved 1/*T*_1_ maps were found to be quite uniform for both samples B and C in both slice orientations. On the other hand, the DDIF rate maps show striking contrast between the two samples. Overall, higher DDIF rates were observed in both the high- and low-resolution image experiments with sample B, which has a higher surface-to-volume ratio than sample C.

**Figure 11:**
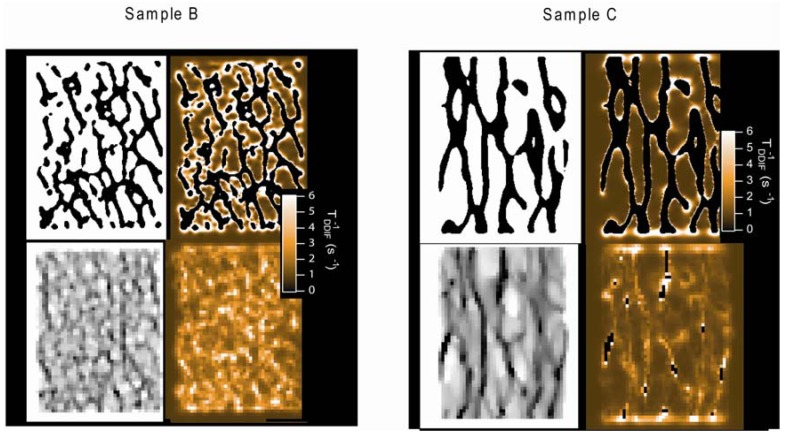
Calculated local DDIF contrast (internal gradient map) of samples B and C. For each sample, the upper left is the binarized high-resolution μCT image. The upper right is the high-resolution calculated DDIF map. The lower left is the downsampled low-resolution image, and the lower right is the calculated low-resolution DDIF map. Reproduced from Reference [24] with permission.

Figure 11 shows the theoretically calculated DDIF decay map of coronal images of both samples based on high-resolution μCT images. These results include binarized high-resolution images and downsampled, partial-volume averaged, low-resolution images. Again, high-resolution DDIF rate maps show pronounced maxima near the surfaces that are transverse to the applied field direction. The coarsening of resolution appears to spread contrast throughout the structure. A qualitative resemblance is clearly observed between the calculated low-resolution DDIF maps and the corresponding experimental DDIF maps, shown in Figure 10.

The *in vitro* studies performed on water-saturated trabecular bone highlight the presence and information content of DDIF contrast in particular biological structures. However, depending on the composition and skeletal location, bone marrow-water diffusion is restricted by adipose cells (which also have low rates of diffusion), which may reduce the achievable DDIF contrast [57,58,59,60]. However, preliminary *in vivo* studies have demonstrated DDIF contrast in human trabecular bone, and studies exploring the influence of marrow composition and/or distribution in the pore space have shed promising light on the *in vivo* source of DDIF contrast [61,62]. Recently, it has been also shown that DDIF contrast is apparent in fresh bone specimens with intact bone marrow [63]. Future work may build upon these results to determine the optimal clinical role of internal field diffusion-weighting in trabecular bone and its potential to infer structural markers of mechanical strength in arbitrary skeletal locations.

### 2.5. Measurement of Magnetic Field Correlation Functions

When the pore size is much smaller than the spatial resolution, statistical description and characterization of the internal magnetic field are compelling alternatives to microimaging. Signal decay due to diffusing spins in the presence of an internal field provide statistical measurements of internal field variation at the microscopic level. Correspondingly, important structural parameters, such as the structural factors of the porous medium, may be approximated by bulk, low-resolution NMR measurements. Audoly *et al*. first calculated the field correlation function of the internal magnetic field of a sample of randomly packed spheres and demonstrated a similarity between the field correlation function and the structural correlation function [27]. This work shows that the statistical features of internal magnetic fields are closely and quantitatively related to the corresponding features of the underlying geometry.

It was recently shown that a pair of symmetric and asymmetric stimulated echo PFG-NMR experiments can be used to directly measure the correlation function of the internal magnetic field [28]. First, we used PFG-NMR to select spins by their translational diffusion displacements without effects from the internal field. Then, a similar experiment was performed that included the effects of the internal field. For a group of spins with specific diffusion displacements, the magnetic field difference across this displacement resulted in additional decay in the second experiment.

**Figure 12:**
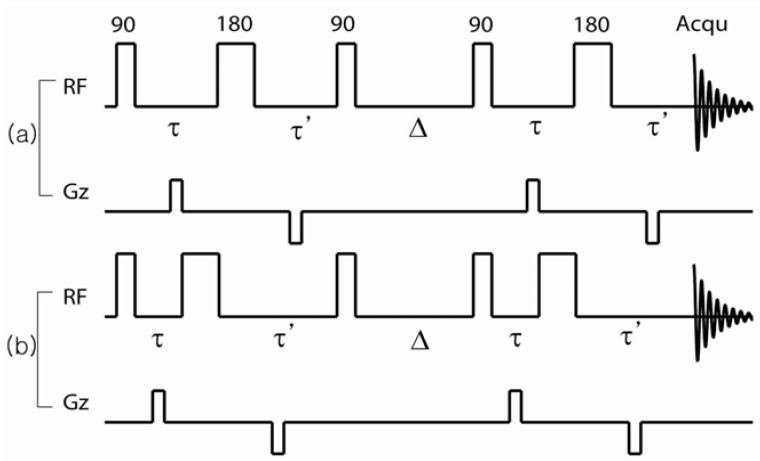
Pulse sequences used to measure the pair correlation function of the internal magnetic field. (**a**) Balanced sequences used to nullify the effect of the internal field; (**b**) Imbalanced sequence used to introduce the effect of the internal field. Reproduced from Reference [28] with permission.

Let us consider a conventional internal field-compensated stimulated echo sequence, $\frac{\pi }{2}-\tau -\pi -\tau^{\prime }-\frac{\pi }{2}-t_{d}-\frac{\pi }{2}-\tau -\pi -\tau^{\prime }$-echo, as shown in Figure 12a. Bipolar external field gradient pulses of length *δ* are applied during the $t_{e}$ periods. The *π*-pulse in the middle of the first and second $\frac{\pi }{2}$-pulses refocuses the phase evolution of the spins due to field inhomogeneities when *τ* = *τ*'. The echo signal of the ensemble of diffusing spins at the echo time is then given by Equation (6).

(6) $$E(\vec{q},\Delta )=\int P(\vec{r},\Delta )e^{i\vec{q}\cdot \vec{r}}d\vec{r}$$

In Equation (5), $\vec{q}=\gamma {\vec{G}}_{\text{ext}}t$ is the wave vector due to the external field gradient. Δ is the diffusion time. A Fourier transform of the echo signal, with respect to $\vec{q}$, will produce the average propagator *P*($\vec{r}$,Δ). In the second experiment, the timing of the *π*-pulse is adjusted so that *τ* − *τ*' = *t* is nonzero; we call this sequence unbalanced, as shown in Figure 12b. In this case, the spins experience the internal field during the unbalanced encoding periods (*τ* ≠ *τ*') causing a net phase accumulation due to the internal field. The echo signal will be further weighted by the internal field effects of the diffusing spins. In this case, the echo signal can be written as shown in Equation (7), within the average propagator formalism.

(7) $$E\prime (\vec{q},\Delta )=\int P(\vec{r},\Delta )e^{i\vec{q}\cdot \vec{r}}e^{i\gamma t(B_{z}^{i}(\vec{r})-B_{z}^{i}(0))}d\vec{r}\equiv \int P\prime (\vec{r},\Delta )e^{i\vec{q}\cdot \vec{r}}d\vec{r}$$

When the full *q*-space is mapped, followed by 3D Fourier transformation, the ratio between the unbalanced and balanced measurements will yield the following.

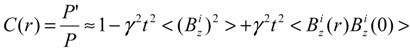
(8)

The second moment ( <${\left(B_{z}^{i}\right)}^{2}$>) and the field correlation function (<$B_{z}^{i}(r)B_{z}^{i}(0)$>) of the susceptibility-induced internal magnetic field ($B_{z}^{i}$) can then be obtained along arbitrary directions of the diffusing spins in the porous medium. The bracket refers to an ensemble average.

For an isotropic sample, a single gradient-direction experiment may be sufficient to measure the propagator because the 3D isotropic propagator can be written as a product of the propagator in each direction. Then, the ratio of the 1D Fourier transformation data between the balanced and unbalanced sequences will yield the following.

(9) $$C1(r_{\|})\equiv \frac{P\prime }{P}\approx b+\gamma^{2}t^{2}<\int P({\vec{r}}_{⊥},\Delta )B_{z}^{i}(\vec{r})B_{z}^{i}(0)d{\vec{r}}_{⊥}>$$

where *b* ≈ 1 − *γ*^2^*t*^2^<${\left(B_{z}^{i}\right)}^{2}$>, and the bracket refers to an ensemble average. Since $\vec{r}$ has two components, parallel to $\left({\vec{r}}_{\|}\right)$ and perpendicular $\left({\vec{r}}_{┴}\right)$ to the applied gradient direction, the integration of ${\vec{r}}_{┴}$ means that *C*1 is only a function of ${\vec{r}}_{\|}$.

For comparison with this type of measurement, numerical evaluation of the internal magnetic field of a densely packed hard sphere model can be performed by the superposition of the magnetic fields from the dipoles located at the center of the each sphere. When a single sphere of radius *R*_0_ and permeability *μ*_1_ is embedded into a medium with permeability *μ*_2_ in the presence of an external magnetic field (${\vec{B}}_{z}$), the magnetic dipole moment ($\vec{m}$) of the sphere due to susceptibility mismatch is given by Equation (9) [64,65,66,67].

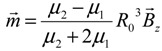
(10)

If we only consider small and isotropic susceptibilities, then the dipole moment along the direction of *B_z_* can be approximated as $\vec{m}=4\pi /3{R_{0}}^{3}\Delta \chi {\vec{B}}_{z}$, where the susceptibility difference between the grain and the medium is 4*π*Δ*χ* = (*μ*_2_ − *μ*_1_). The internal magnetic field at position $\vec{R}$ is given by the superposition of the dipole fields of each sphere and can be calculated as show in Equation (11).

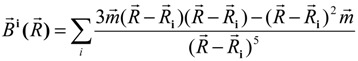
(11)

In Equation (11), ${\vec{R}}_{\text{i}}$ is the center of each sphere. As before, if we take the *B*_0_-field along the *z*-direction, the *z*-component of the internal field is the only relevant component of the NMR experiment.

Now, the correlation function of the internal magnetic field is analogous in form to that derived from a single PFG direction experiment. Specifically, the magnetic field correlation function, derived from the calculated internal field distribution is described by Equation (12).

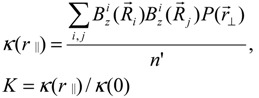
(12)

In Equation (12), $|{\vec{R}}_{i}-{\vec{R}}_{j}|=r_{\|}$ ($r_{\|}$ is the direction of the applied field gradient), *n*' is the number of coordinate pairs with distance $r_{\|}$, and ${\vec{r}}_{┴}$ is the component of ${\vec{R}}_{i}-{\vec{R}}_{j}$ that is perpendicular to the direction of applied gradient. P(${\vec{r}}_{┴}$) is the probability function that weights each contribution to the sum by the number of coordinate pairs whose projected separation perpendicular to the field gradient direction is ${\vec{r}}_{┴}$. In that sense *P* is similar to an experimentally measured propagator but in this expression is evaluated purely numerically.

**Figure 13:**
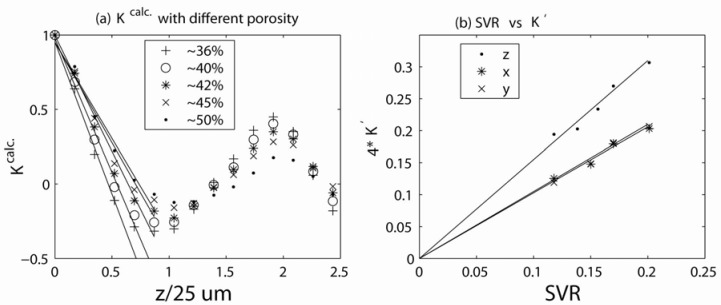
(**a**) Numerically evaluated pair correlation functions of the internal fields with varying porosity; (**b**) Linear relationship between the initial slope of the pair correlation function and surface-to-volume ratio obtained by the porosity of the pack [68]. Reproduced from Reference [28] with permission.

Figure 13a shows the numerically calculated internal field correlations (*K*(*r*_‖_), *r*_‖_ // *z*), where *z* is the direction of the main magnetic field) as a function of the porosity of a random packing of spheres. The porosity of the bead pack is changed by randomly removing beads from the pack. The initial slope decreases as the porosity increases, which also reduces the surface-to-volume ratio. Figure 13b shows a linear relationship between the initial slope of the calculated field correlation function and the surface-to-volume ratio of the system of packed spheres, which is consistent with previously reported findings (*K*'(*r*_‖_) = *A*/4*SVR) [27]. On the other hand, strong anisotropy was observed when the direction of *r*_‖_ was changed in the calculation. The slope A was found to be 1.5, 1.1, and 1.1 along *r*_‖_ = *z*,*x*, and *y*, respectively. Audoly *et al*., reported A = 1.28 when *r*_‖_ = *r* (*i.e*., the average of the 3D ensemble), which corresponds to the average value of the three orthogonal A values in the internal field correlation function.

**Figure 14:**
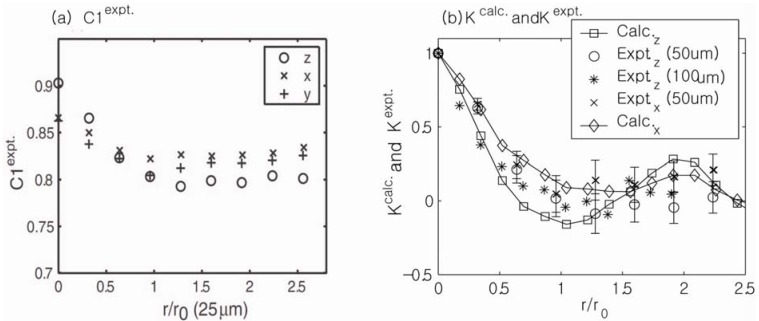
(**a**) Experimental measurements of the field correlation functions of a randomly packed sample of glass beads. Three independent measurements along the *r*_‖_ = *z*,*x*,*y* directions of external gradient are shown. The reduction in [*C*_1_(0) − *C*_1_(∞)] was strongest along the *z*-direction. (**b**) Comparisons of the experimental measurements with the numerical calculations. Reproduced from Reference [28] with permission.

Figure 14a shows experimental measurements of the field correlation function of a sample of randomly packed glass beads using the unbalanced stimulated echo technique. Three independent measurements along the *r*_‖_ = *z*,*x*,*y* directions of the external gradient are shown. The reduction in [*C*_1_(0) − *C*_1_(∞)] was strongest along the *z*-direction (the direction of the main magnetic field), and this reduction was similarly smaller along the *x*- and *y*-directions in the gradient. The anisotropy ratio of the reduction in the experimental measurements (2.2:0.92:1) was also in good agreement with the theoretical calculations of *κ*(0) (1.8:0.97:1) along *z*, *x* and *y* directions. 

Comparison between the numerically and experimentally measured field correlation functions (*K* = [*C*1(*r*_‖_) − *b*]/[*C*1(0) − *b*]) for different bead sizes and gradient directions are shown in Figure 14b. The surface-to-volume ratios extracted from the initial slopes of the field correlation function measurements were in generally good agreement with the surface-to-volume ratios calculated in separate porosity measurements. Full 3D *q*-space mapping is expected to yield a complete description of the correlation function of internal magnetic fields along arbitrary directions and resolve the residual discrepancies between theory and experiment in Figure 14. Because the area of comprehensive 3D susceptibility mapping is becoming quite important to the *in vivo* clinical realm [65,66,67], the proposed correlation function mapping may have diagnostic potential.

## 3. Conclusions

Susceptibility contrast is ubiquitous in materials, and MR is uniquely sensitive to magnetic field variations caused by susceptibility contrast. Characterization of the internal magnetic field properties of porous media and the development of new, relevant MR pulse sequences have the potential to provide important structural and functional information regarding porous media via nondestructive techniques.

This paper reviews the basic concept of DDIF contrast and its extension to imaging. Appropriate data-analysis methods for bulk- and DDIF-imaging experiments have been discussed. DDIF-imaging experiments with 2D cylindrical phantoms show the direct correlation between DDIF contrast and the strength of the local internal gradients due to susceptibility differences. Interpretation of bulk-DDIF contrast in trabecular bone samples was confirmed using both micro-imaging experiments and internal field calculations of the same samples. A new PFG-NMR method for measuring magnetic field correlations was presented, and representative experiments and theoretical calculations are described for a randomly packed system of spheres, showing the potential for utilizing the statistical features of internal fields to obtain structural information. Potential applications of this method for use in *in vivo* trabecular bone and microvasculature imaging are in progress.

## References

[1] Brown R.J. (1961). Distribution of fields from randomly placed dipoles—Free-precession signal decay as result of magnetic grains. Phys. Rev..

[2] Williams W.D., Seymour E.F.W., Cotts R.M. (1978). Pulsed-gradient multiple-spin-echo NMR technique for measuring diffusion in presence of background magnetic-field gradients. J. Magn. Reson..

[3] Seland J.G., Sorland G.H., Zick K., Hafskjold B. (2000). Diffusion measurements at long observation times in the presence of spatially variable internal magnetic field gradients. J. Magn. Reson..

[4] Sorland G.H., Hafskjold B., Herstad O. (1997). A stimulated-echo method for diffusion measurements in heterogeneous media using pulsed field gradients. J. Magn. Reson..

[5] Sun P.Z., Seland J.G., Cory D. (2003). Background gradient suppression in pulsed gradient stimulated echo measurements. J. Magn. Reson..

[6] Cotts R.M., Hoch M.J.R., Sun T., Markert J.T. (1989). Pulsed field gradient stimulated echo methods for improved NMR diffusion measurements in heterogeneous systems. J. Magn. Reson..

[7] Lisitza N.V., Song Y.Q. (2002). Manipulation of the diffusion eigenmodes in porous media. Phys. Rev. B.

[8] Song Y.Q. (2001). Pore sizes and pore connectivity in rocks using the effect of internal field. Magn. Reson. Imaging.

[9] Song Y.Q., Ryu S., Sen P.N. (2000). Determining multiple length scales in rocks. Nature.

[10] Mitra P.P., Sen P.N., Schwartz L.M. (1993). Short-time behavior of the diffusion-coefficient as a geometrical probe of porous-media. Phys. Rev. B.

[11] Sen P.N., Schwartz L.M., Mitra P.P. (1994). Probing the structure of porous-media using NMR spin echoes. Magn. Reson. Imaging.

[12] Sigmund E.E., Cho H., Chen P., Byrnes S., Song Y.Q., Guo X.E., Brown T.R. (2008). Diffusion-based MR methods for bone structure and evolution. Magn. Reson. Med..

[13] Shen T., Weissleder R., Papisov M., Bogdanov A., Brady T.J. (1993). Monocrystalline iron-oxide nanocompounds (mion)—Physicochemical properties. Magn. Reson. Med..

[14] Hahn P.F., Saini S., Weissleder R., Mayosmith W.W. (1995). Superparamagnetic iron-oxide contrast agents in abdominal MR-imaging. Radiology.

[15] Weissleder R., Bogdanov A., Neuwelt E.A., Papisov M. (1995). Long-Circulating iron-oxides for MR imaging. Adv. Drug. Deliv. Rev..

[16] Bogdanov A., Weissleder R., Brady T.J. (1995). Long-circulating blood-pool imaging agents. Adv. Drug Deliv. Rev..

[17] Dennie J., Mandeville J.B., Boxerman J.L., Packard S.D., Rosen B.R., Weisskoff R.M. (1998). NMR imaging of changes in vascular morphology due to tumor angiogenesis. Magn. Reson. Med..

[18] Pathak A.P., Ward B.D., Schmainda K.M. (2008). A novel technique for modeling susceptibility-based contrast mechanisms for arbitrary microvascular geometries: The finite perturber method. Neuroimage.

[19] Pathak A.P., Rand S.D., Schmainda K.M. (2003). The effect of brain tumor angiogenesis on the *in vivo* relationship between the gradient-echo relaxation rate change (Delta R2*) and contrast agent (MION) dose. J. Magn. Reson. Imaging.

[20] Kiselev V.G. (2001). On the theoretical basis of perfusion measurements by dynamic susceptibility contrast MRI. Magn. Reson. Med..

[21] Jensen J.H., Chandra R., Ramani A., Lu H.Z., Johnson G., Lee S.P., Kaczynski K., Helpern J.A. (2006). Magnetic field correlation imaging. Magn. Reson. Med..

[22] Jensen J.H., Szulc K., Hu C.X., Ramani A., Lu H.Z., Xuan L., Falangola M.F., Chandra R., Knopp E.A., Schenck J. (2009). Magnetic field correlation as a measure of iron-generated magnetic field inhomogeneities in the brain. Magn. Reson. Med..

[23] Cho H., Ryu S., Ackerman J.L., Song Y.Q. (2009). Visualization of inhomogeneous local magnetic field gradient due to susceptibility contrast. J. Magn. Reson..

[24] Sigmund E.E., Cho H., Song Y.Q. (2009). High-resolution MRI of internal field diffusion-weighting in trabecular bone. Nmr. Biomed..

[25] Lisitza N.V., Song Y.Q. (2001). The behavior of diffusion eigenmodes in the presence of internal magnetic field in porous media. J .Chem. Phys..

[26] Pomerantz A.E., Song Y.Q. (2008). Quantifying spatial heterogeneity from images. New. J. Phys..

[27] Audoly B., Sen P.N., Ryu S., Song Y.Q. (2003). Correlation functions for inhomogeneous magnetic field in random media with application to a dense random pack of spheres. J. Magn. Reson..

[28] Cho H., Song Y.Q. (2008). NMR measurement of the magnetic field correlation function in porous media. Phys. Rev. Lett..

[29] Stejskal E.O., Tanner J.E. (1965). Spin diffusion measurements: Spin echoes in the presence of a time-dependent field gradient. J. Chem. Phys..

[30] Callaghan P.T. (1991). Principles of Nuclear Magnetic Resonance Microscopy.

[31] Basser P.J., Mattiello J., Lebihan D. (1994). Estimation of the effective self-diffusion tensor from the NMR spin-echo. J. Magn. Reson. B.

[32] Mori S., Crain B.J., Chacko V.P., van Zijl P.C.M. (1999). Three-dimensional tracking of axonal projections in the brain by magnetic resonance imaging. Ann. Neurol..

[33] Mitra P.P., Sen P.N., Schwartz L.M., Ledoussal P. (1992). Diffusion propagator as a probe of the structure of porous-media. Phys. Rev. Lett..

[34] Hurlimann M.D., Latour L.L., Sotak CH. (1994). Diffusion measurement in sandstone core—NMR determination of surface-to-volume ratio and surface relaxivity. Magn. Reson. Imaging..

[35] Song Y.Q. (2000). Determining pore sizes using an internal magnetic field. J. Magn. Reson..

[36] Sun B.Q., Dunn K.J. (2002). Probing the internal field gradients of porous media. Phys. Rev. E.

[37] Zhong J.H., Kennan R.P., Gore J.C. (1991). Effects of susceptibility variations on NMR measurements of diffusion. J. Magn. Reson..

[38] Chen Q., Gingras M.K., Balcom B.J. (2003). A magnetic resonance study of pore filling processes during spontaneous imbibition in Berea sandstone. J. Chem. Phys..

[39] Yablonskiy D.A., Haacke E.M. (1994). Theory of NMR signal behavior in magnetically inhomogeneous tissues—The static dephasing regime. Magn. Reson. Med..

[40] Reichenbach J.R., Venkatesan R., Yablonskiy D.A., Thompson M.R., Lai S., Haacke E.M. (1997). Theory and application of static field inhomogeneity effects in gradient-echo imaging. J. Magn. Reson. Imaging.

[41] Hahn E.L. (1950). Spin echoes. Phys. Rev..

[42] Kimmich R., Unrath W., Schnur G., Rommel E. (1991). NMR measurement of small self-diffusion coefficients in the fringe-field of superconducting magnets. J. Magn. Reson..

[43] Han S.H., Song Y.K., Cho F.H., Ryu S., Cho G., Song Y.Q., Cho H. (2011). Magnetic field anisotropy based MR tractography. J. Magn. Reson..

[44] Palombo M., Gabrielli A., De Santis S., Cametti C., Ruocco G., Capuani S. (2011). Spatio-temporal anomalous diffusion in heterogeneous media by nuclear magnetic resonance. J. Chem. Phys..

[45] Chung H., Wehrli F.W., Williams J.L., Kugelmass S.D. (1993). Relationship between NMR transverse relaxation, trabecular bone architecture, and strength. Proc. Natl. Acad. Sci. USA.

[46] Chung H.W., Wehrli F.W., Williams J.L., Wehrli S.L. (1995). 3-Dimensional nuclear-magnetic-resonance microimaging of trabecular bone. J. Bone Miner. Res..

[47] Goldstein S.A., Goulet R., Mccubbrey D. (1993). Measurement and significance of 3-dimensional architecture to the mechanical integrity of trabecular bone. Calcified Tissue Int..

[48] Kleerekoper M., Villanueva A.R., Stanciu J., Rao D.S., Parfitt A.M. (1985). The role of 3-dimensional trabecular microstructure in the pathogenesis of vertebral compression fractures. Calcified Tissue Int..

[49] Ma J.F., Wehrli F.W. (1996). Method for image-based measurement of the reversible and irreversible contribution to the transverse-relaxation rate. J. Magn. Reson. B.

[50] Newitt D.C., Majumdar S., Jergas M.D., Genant H.K. (1996). Decay characteristics of bone marrow in the presence of a trabecular bone network: *In vitro* and *in vivo* studies showing a departure from monoexponential behavior. Magn. Reson. Med..

[51] Wehrli F.W., Song H.K., Saha P.K., Wright A.C. (2006). Quantitative MRI for the assessment of bone structure and function. NMR Biomed..

[52] Saha P., Benito M., Vasilic B., Snyder P., Wehrli F.W. (2006). Tensor-scale measures obtained by *in vivo* micro-MRI detects increased trabecular bone anisotropy in hypogonadal men. J. Bone Miner. Res..

[53] Phan C.M., Matsuura M., Bauer J.S., Dunn T.C., Newitt D., Lochmueller E.M., Eckstein F., Majumdar S., Link T.M. (2006). Trabecular bone structure of the calcaneus: Comparison of MR imaging at 3.0 and 1.5 T with micro-CT as the standard of reference. Radiology.

[54] Cho H., Baugh J., Ryan C.A., Cory D.G., Ramanathan C. (2007). Low temperature probe for dynamic nuclear polarization and multiple-pulse solid-state NMR. J. Magn. Reson..

[55] Friedrich K.M., Chang G., Vieira R.L.R., Wang L.G., Wiggins G.C., Schweitzer M.E., Regatte R.R. (2009). *In vivo* 7.0-tesla magnetic resonance imaging of the wrist and hand: technical aspects and applications. Semin. Musculoskel. R..

[56] Krug R., Stehling C., Kelley D.A.C., Majumdar S., Link T.M. (2009). Imaging of the musculoskeletal system *in vivo* using ultra-high field magnetic resonance at 7 t. Invest Radiol..

[57] Oner A.Y., Aggunlu L., Akpek S., Tali T., Celik A. (2007). Diffusion-weighted imaging of the appendicular skeleton with a non-Carr-Purcell-Meiboom-Gill single-shot fast spin-echo sequence. Am. J. Roentgenol..

[58] Ueda Y., Miyati T., Ohno N., Motono Y., Hara M., Shibamoto Y., Kasai H., Kawamitsu H., Matsubara K. (2010). Apparent diffusion coefficient and fractional anisotropy in the vertebral bone marrow. J. Magn. Reson. Imaging.

[59] Dietrich O., Biffar A., Reiser M.F., Baur-Melnyk A. (2009). Diffusion-weighted imaging of bone marrow. Semin. Musculoskel. R..

[60] Ababneh Z.Q., Beloeil H., Berde C.B., Ababneh A.M., Maier S.E., Mulkern R.V. (2009). *In vivo* lipid diffusion coefficient measurements in rat bone marrow. Magn. Reson. Imaging.

[61] Sigmund E., Regatte R., Schweitzer M., Cho H., Song Y.-Q. (2009). *In vivo* imaging of signal decay due to diffusion in the internal field in human knee trabecular bone. Diffus. Fundam..

[62] De Santis S., Rebuzzi M., di Pietro G., Fasano F., Maraviglia B., Capuani S. (2010). *In vitro* and *in vivo* MR evaluation of internal gradient to assess trabecular bone density. Phys. Med. Biol..

[63] Mintzopoulos D., Ackerman J.L., Song Y.Q. (2011). MRI of trabecular bone using a decay due to diffusion in the internal field contrast imaging sequence. J. Magn. Reson. Imaging.

[64] Jackson J.D. (1998). Classical Electrodynamics.

[65] Liu C.L. (2010). Susceptibility tensor imaging. Magn. Reson. Med..

[66] Deistung A., Rauscher A., Sedlacik J., Stadler J., Witoszynskyj S., Reichenbach J.R. (2008). Susceptibility weighted imaging at ultra high magnetic field strengths: theoretical considerations and experimental results. Magn. Reson. Med..

[67] Miller K.L. (2010). Asymmetries of the balanced ssfp profile. Part I: Theory and observation. Magn. Reson. Med..

[68] Latour L.L., Mitra P.P., Kleinberg R.L., Sotak C.H. (1993). Time-dependent diffusion-coefficient of fluids in porous-media as a probe of surface-to-volume ratio. J. Magn. Reson. A.

